# The third-generation EGFR inhibitor AZD9291 overcomes primary resistance by continuously blocking ERK signaling in glioblastoma

**DOI:** 10.1186/s13046-019-1235-7

**Published:** 2019-05-23

**Authors:** Xuejiao Liu, Xiangyu Chen, Lin Shi, Qianqian Shan, Qiyu Cao, Chenglong Yue, Huan Li, Shengsheng Li, Jie Wang, Shangfeng Gao, Mingshan Niu, Rutong Yu

**Affiliations:** 1 Insititute of Nervous System Diseases, Affiliated Hospital of Xuzhou Medical University, Xuzhou Medical University, Xuzhou, Jiangsu China; 2 Department of Neurosurgery, Affiliated Hospital of Xuzhou Medical University, Xuzhou, Jiangsu China; 3 Blood Diseases Institute, Xuzhou Medical University, Xuzhou, Jiangsu China; 4 Surgical Department 9, Xuzhou children’s hospital, Xuzhou, Jiangsu China

**Keywords:** GBM, AZD9291, EGFR/ERK signaling pathway, Cell proliferation

## Abstract

**Background:**

Glioblastoma (GBM) is a fatal brain tumor, lacking effective treatment. Epidermal growth factor receptor (EGFR) is recognized as an attractive target for GBM treatment. However, GBMs have very poor responses to the first- and second-generation EGFR inhibitors. The third-generation EGFR-targeted drug, AZD9291, is a novel and irreversible inhibitor. It is noteworthy that AZD9291 shows excellent blood–brain barrier penetration and has potential for the treatment of brain tumors.

**Methods:**

In this study, we evaluated the anti-tumor activity and effectiveness of AZD9291 in a preclinical GBM model.

**Results:**

AZD9291 showed dose-responsive growth inhibitory activity against six GBM cell lines. Importantly, AZD9291 inhibited GBM cell proliferation > 10 times more efficiently than the first-generation EGFR inhibitors. AZD9291 induced GBM cell cycle arrest and significantly inhibited colony formation, migration, and invasion of GBM cells. In an orthotopic GBM model, AZD9291 treatment significantly inhibited tumor survival and prolonged animal survival. The underlying anti-GBM mechanism of AZD9291 was shown to be different from that of the first-generation EGFR inhibitors. In contrast to erlotinib, AZD9291 continuously and efficiently inhibited the EGFR/ERK signaling in GBM cells.

**Conclusion:**

AZD9291 demonstrated an efficient preclinical activity in GBM in vitro and in vivo models*.* AZD9291 has been approved for the treatment of lung cancer with good safety and tolerability. Our results support the possibility of conducting clinical trials of anti-GBM therapy using AZD9291.

**Electronic supplementary material:**

The online version of this article (10.1186/s13046-019-1235-7) contains supplementary material, which is available to authorized users.

## Background

Glioblastoma (GBM) is the most common malignant adult brain tumor and has the poorest prognosis of all brain tumors in adults [1]. To date, GBM treatment is based on surgical maximum excision combined with postoperative radiotherapy and chemotherapy [2]. Although GBM diagnosis and treatment techniques have been greatly improved, the median survival of GBM patients is still only 15–23 months, with less than a 6% five-year survival rate [3]. Hence, there is an urgent need to develop new therapeutic drugs to treat GBM.

Epidermal growth factor receptor (EGFR) is a transmembrane receptor tyrosine kinase. Abnormal expression of EGFR regulates tumor cell proliferation, migration, differentiation, and homeostasis [4]. Approximately 57% of GBMs have EGFR genetic variants, including mutations, rearrangements, selective splicing, and amplifications [5, 6]. Unlike lung cancer, with EGFR mutations mainly in the kinase domain, GBM exhibits EGFR mutations mainly in the extracellular domain [7]. EGFR mutations enable GBM cells to activate downstream PI3K/AKT and RAS/ERK signaling pathways independent of EGF ligands. Numerous studies have confirmed that EGFR overexpression and mutation promote the growth and survival of GBM [8, 9]. Knockout of the *EGFR* gene have confirmed that the survival of *EGFR-*mutant GBM cells is dependent on EGFR function [7]. Therefore, EGFR has long been considered a very attractive target for the treatment of GBM.

Many studies have targeted EGFR for GBM treatment, but so far no therapeutic effect has been reported [10, 11]. The first-generation EGFR inhibitors, such as gefitinib and erlotinib, and the second-generation EGFR inhibitor afatinib have been demonstrated to inhibit GBM cell growth, proliferation, and angiogenesis. However, these EGFR inhibitors have not shown therapeutic efficacy in clinical trials. Gefitinib did not improve overall survival of the patients in a phase II trial of relapsed GBM or in phase I/II trials of newly diagnosed GBM combined with radiotherapy [12]. Erlotinib did not show any therapeutic efficacy and caused unacceptable side effects in GBM patients [13]. Subsequent clinical trials evaluating the therapeutic effects of combined erlotinib and rapamycin (an mTOR inhibitor) or combined erlotinib and bevacizumab on recurrent GBM were also unsuccessful [14]. In addition, the second-generation EGFR inhibitor afatinib showed no effective outcomes in clinical trials for the treatment of primary or recurrent GBM [15]; these trials confirmed that afatinib barely penetrated the blood-brain barrier. Two main explanations for the failure of the above EGFR inhibitors in GBM treatment have been reached [16]. First, relatively high concentrations of the above EGFR inhibitors are required to inhibit GBM cell proliferation in vitro*.* Achieving such high drug concentrations in the brain is a great challenge. Second, the abilities of these four EGFR inhibitors to cross the blood-brain barrier are very poor. Therefore, selection of an EGFR inhibitor with better activity and ability to penetrate through the blood-brain barrier will allow more rational and targeted design in anti-GBM therapy.

Osimertinib (AZD9291) is an oral, irreversible, third-generation EGFR inhibitor [17]. AZD9291 has been marketed for the treatment of lung cancer with very good therapeutic effects [18]. The ability of drugs to penetrate through the blood-brain barrier is one of the key factors in determining the therapeutic efficacy of brain tumors. P-glycoprotein (P-gp) and breast cancer resistance protein (BCRP) transporters are important in blocking the passage of various molecules across the blood-brain barrier [19]. Unlike the chemical structures of other EGFR tyrosine kinase inhibitors (EGFR-TKIs), AZD9291 is a substrate for P-gp and BCRP and thus easily penetrates through the blood-brain barrier [20]. Study of an animal model has demonstrated that AZD9291 penetrates well and passes through the blood–brain barrier, and is 5–25 times more concentrated in brain tissue than in plasma [21]. In addition, AZD9291 in brain tissue can reach a concentration approximately 10-fold higher than gefitinib can. Compared to other EGFR inhibitors, AZD9291 has shown a good ability to inhibit tumor cell growth in a mouse model with brain metastases of lung cancer. AZD9291 effectively eliminates lung cancer cells which have metastasized to the brain of patients in clinical study [20]. AZD9291 targets cysteine-797 residue in the ATP binding site of intracellular tyrosine kinase domain with T790 M mutation to exert its anti-cancer effect in lung cancer [22]. However, AZD9291 can still inhibit the kinase activity of wild-type EGFR with weaker binding than T790 M mutant EGFR (IC_50_: 184 vs 1 nM) [21]. GBM exhibits EGFR mutations mainly in the extracellular domain of EGFR. In contrast, the intracellular kinase domain of EGFR remains wild-type in GBM. Thus, AZD9291 may inhibit the activity of EGFR in GBM through blocking the function of intracellular kinase domain. In short, AZD9291 may be a suitable EGFR inhibitor for the treatment of GBM.

This study evaluated the effects of AZD9291 on GBM cell proliferation, colony formation, migration, and invasion, as well as the anti-GBM therapeutic efficacy of AZD9291 in a mouse intracranial GBM model. This preclinical study provides support for clinical trials of AZD9291 in GBM treatment.

## Materials and methods

### Cell lines and reagents

Human GBM cell lines U87, U251, U118, LN229, T98G and LN18 were cultured in Dulbecco’s modified Eagle’s medium (DMEM) supplemented with 10% FBS. These cell lines were grown in a humidified incubator containing 5% CO_2_ at 37 °C. AZD9291 and PD098059 were purchased from MedChem Express (Guangzhou, China). ERK inhibitor SCH772984 was obtained from CSNpharm (CSN13643, CSNpharm, Chicago, USA). EGF was purchased from RD systems. EGFR, p-EGFR (Tyr1068), p-ERK (Thr202/Tyr204), p-AKT (Thr473), p-STAT3 (Tyr705), cleaved caspase-3 and β-actin primary antibodies were purchased from Cell Signaling Technology. Primary antibody against Ki67 were obtained from Thermo Scientific. DAPI was purchased from Sigma-Aldrich.

### Construction of EGFR-WT and EGFR-C797S stable cell lines

The cDNA encoding human EGFR-WT or EGFR with the C797S mutation was inserted into the pCDH-513B-1 lentiviral vector. The viruses were produced in 293FT cells by co-transfecting the recombinant plasmids with the helper plasmids pSPXA2 and pMD2.G. The U87 cells were then transfected with the EGFR-WT or EGFR with the C797S lentivirus for 48 h and then continuously cultured in medium containing 2.5 μg/mL puromycin. The surviving cells were cultured and used to generate cell lines that stably expressed EGFR gene.

### Cell viability assay

For the viability assays [23], the GBM cell lines were plated in 96-well plates at a density of 3000 cells per well and allowed to adhere for 24 h. The cells were treated with 0.1% dimethyl sulfoxide (DMSO) or different doses (0–20 μM) of AZD9291 for 72 h. Then 10 μL of CCK8 was added to each well and incubated for 2 h. The absorbance was measured at 490 nm in a microplate reader. The experiment was repeated three times.

### EdU incorporation assay

A Cell-Light™ EdU Cell Proliferation Detection Kit (Ruibo Biotech, Guangzhou, China) was used to detect cell proliferation in accordance with the manufacturer’s instructions [24]. U87 and U251 cells were seeded in 96-well plates. After the cells were attached to the plate, they were treated with 0.1% DMSO or different concentrations (0.5, 1 and 2 μM) of AZD9291. After 24 h, 50 μΜ EdU was added, and then the cells were incubated for another 4 h. This was followed by fixing with 4% paraformaldehyde for 15 min and treating with 0.5% Triton X-100 for 20 min. The cells were incubated with 1 × Apollo® reaction cocktail for 30 min and then stained with DAPI for 15 min. After three washes in phosphate-buffer saline (PBS), the cells were observed and photographed with an inverted fluorescence microscope (Olympus, Japan). This experiment was repeated three times.

### Two dimensional (2D) and three (3D) dimensional colony forming assay

The U87 and U251 cells were seeded in 6-well plates with 500 cells per well and three replicated wells in each group. After the cells were attached to the well, the cells in the experimental group were treated with AZD9291 at indicated concentrations (0.5, 1 and 2 μM). After 24 h of treatments, the media were changed, and the cells were further incubated with fresh drug-free medium for 10–14 d. The cells were then washed with PBS and fixed with methanol, followed by staining with a 0.1% crystal violet solution. After rinsing off the dye, the cells were observed, photographed, and counted under a microscope.

The ability to create colonies was further verified using 3D culture with methylcellulose. Frozen methylcellulose containing medium (2% stock solution) was thawed at 4 °C. Complete methylcellulose medium was prepared by mixing 3 mL of 2.0% methylcellulose stock with 3 mL of DMEM medium with 10% final concentration of fetal bovine serum. Cells were added to complete methylcellulose medium at a density of 600 cells/mL and mixed well. Cells were plated in the culture mixture in 6-well plates (three per group) and incubated at 37 °C in a humidified atmosphere with 5% CO_2_ for 13–16 days.

### Cell cycle analysis

Flow cytometry was used in this study to detect the cell cycle distribution in GBM cells after AZD9291 treatment. The U87 and U251 cells were plated in 6-cm culture dishes. Once the cells were adhered to the dish, 2 μΜ AZD9291 was added, and the cells were further incubated for 24 h. The harvested cells were fixed with 70% ice-cold ethanol and subsequently washed twice with PBS. A staining solution containing 50 μg/mL propidium iodide (PI) solution and 25 μg/mL ribonuclease (RNase) were added to the cells for 30 min. Subsequently, the cells were assayed on a FACSCalibur (Becton-Dickinson) and analyzed by CellQuest Pro software (Becton-Dickinson).

### Cell migration assay

The U87 and U251 cells were plated in 6-well plates and cultured overnight. Once the cells reached 90% confluence, a plastic pipette tip was used to scratch a wound in the monolayer of cells. After washing in PBS, the media was replaced with serum-free medium containing either 0.1% DMSO or different concentrations of AZD9291. Cells were then cultured for 24 or 48 h, followed by randomly selecting five fields of view at the edge of the disrupted area to photograph under a microscope. This experiment was performed three times. The numbers of cells migrating in the scratch were counted in each treatment group for statistical analysis.

### Transwell invasion assay

Cell invasion was detected using a transwell system according to our previous report [24]. In brief, 50 μL Matrigel with a final concentration of 1 mg/mL was applied to the transwell chamber. The U87 and U251 cells cultured with serum-free medium containing either 0.1% DMSO or AZD9291 at indicated concentrations were added to the upper layer of the transwell chamber. DMEM medium containing 10% FBS was added to the lower transwell chamber to incubate the cells for 30 h. Subsequently, the cells that invaded to the lower transwell chamber were stained with 0.1% crystal violet for 5 min. Five fields of these cells were randomly selected for photographing and counting under a microscope to analyze the cell invasion in each group.

### In vivo studies

Protocols for animal experiments in this study were approved by the ethics committee of Xuzhou Medical University. We purchased 5–6-week old male BALB/c athymic nude mice from Weitong Lihua Experimental Animal Technology Co., Ltd. (Beijing, China). U87 cells (5 × 10^5^ cells per mouse) were injected intracranially into the right striatum of these mice using a small animal stereotaxic apparatus [25]. Five days after the tumor cell inoculation, the nude mice bearing tumor cells were randomly divided into three groups (*n* = 14 mice per group). Each group of mice was treated as follows: the control group was intraperitoneally injected or oral administration with vehicle once every other day, and the drug treatment groups were intraperitoneally injected or oral administration with low-dose (15 mg/kg) or high-dose (30 mg/kg) AZD9291 once every other day. After 25 d of treatment, five mice from each group were randomly selected and euthanized. The tumors of the control and treated mice were harvested and prepared for histological study. The remaining nine mice in each group were used for survival analysis.

### Histopathology and immunofluorescence staining

The dissected whole brains of the control and the drug treatment groups were fixed in 4% paraformaldehyde for 24 h, followed by continuously dehydrating in 20 and 30% sucrose solutions until the whole brains sank to the bottom of the container. The whole brains were dehydrated in each of the gradient solutions for 24 h. The frozen glioma tissues were serially sectioned at a thickness of 12 μm. The brain slices were then stained with hematoxylin and eosin (H&E) solutions and subsequently were observed. We photographed the tumors under a light microscope to determine their sizes.

The remaining brain slices with tumors were incubated with PBS containing 0.3% Triton X-100 at room temperature for 30 min. We then directly added blocking solution containing 10% goat serum and incubated the slices for 1 h. Subsequently, anti-Ki67 and anti-cleaved caspase-3 primary antibodies were independently added to the brain slices, and the slices were incubated overnight at 4 °C. After the slices were washed, the corresponding secondary antibodies were applied to the brain slices and incubated for 1 h at room temperature in the dark. The brain slices were stained with DAPI solution to identify the nuclei. The results of the fluorescent staining were observed and photographed with an inverted fluorescence microscope.

### Western blot analysis

U87 and U251 cells were incubated with different concentrations of AZD9291 for 24 h. Total protein were extracted and protein concentrations were determined by the Bradford method. Then, fifty micrograms of total sample proteins were run on 10% polyacrylamide gels and electrotransferred to polyvinylidene difluoride (PVDF) membranes. Subsequently, membranes were blocked with 5% defatted milk powder for 1 h at room temperature, incubated with indicated antibodies at 4 °C overnight, and then were incubated with secondary antibodies at room temperature for 2 h. The signals were detected by the ECL detection system.

### Statistical analysis

Each experiment was repeated more than 3 times independently. Statistical analyses were performed using the SPSS Version 16.0. The data were presented as the means ± SEM of three to five independent experiments. Differences between control and treatment groups were calculated using the unpaired t-test. A Kaplan-Meier survival curve was used for the survival analysis. *P* values < 0.05 was considered as statistically significant.

## Results

### AZD9291 inhibits GBM cell proliferation

**Fig. 1:**
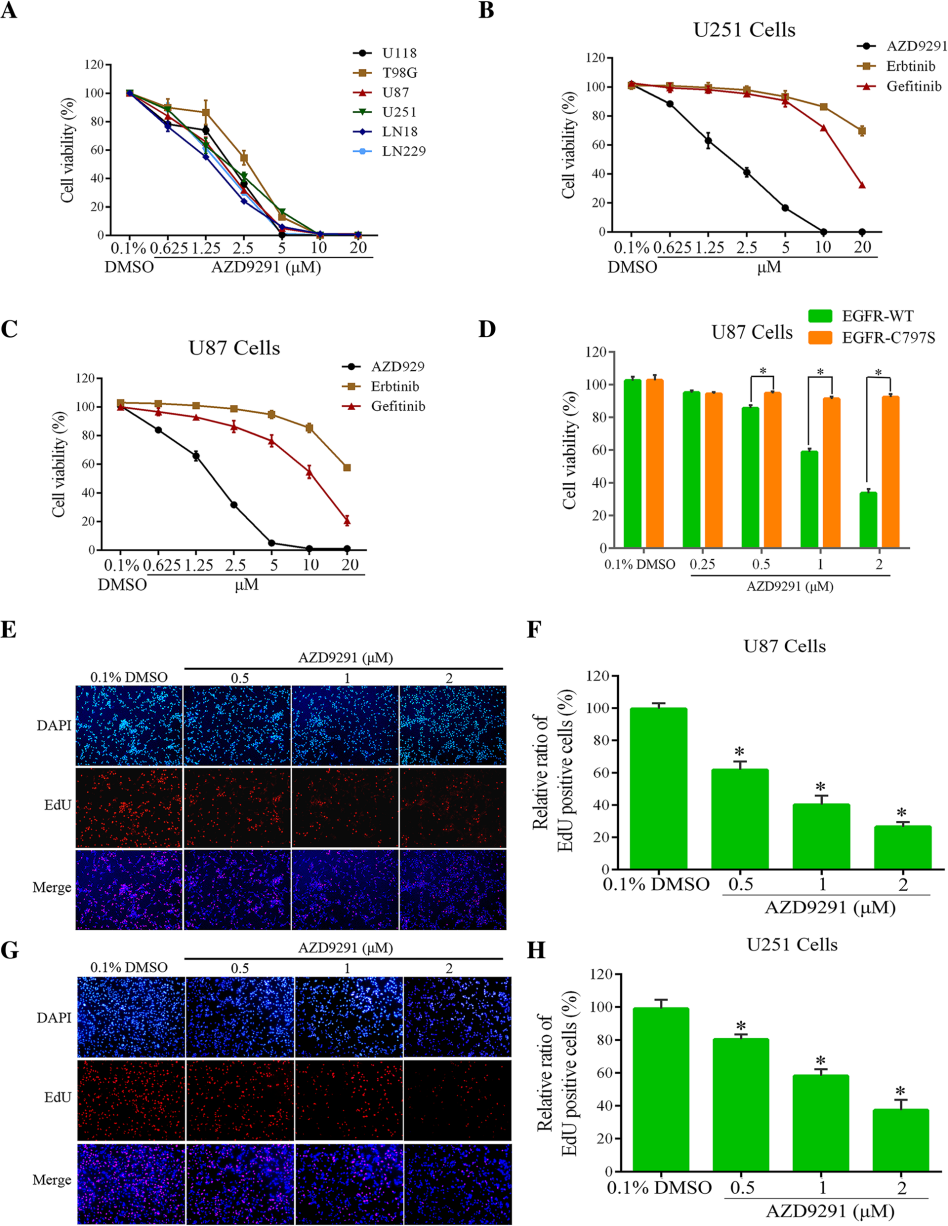
AZD9291 inhibits GBM cell viability and proliferation. **a** GBM cells were treated by different concentrations of AZD9291 for 72 h. The cell viability was examined by CCK8 assay. Comparation of different sensitive of GBM cells to EGFR inhibitors (AZD9291, Erlotinib and Gefitinib). U251 (**b**) and U87 (**c**) cells were incubated with indicated concentrations of AZD9291, Erlotinib and Gefitinib for 72 h, respectively. Cell viability was assessed by CCK8 assay. **d** Mutation of EGFR abolishes the inhibitory activity of AZD9291 in GBM cells. EGFR WT and EGFR C797S mutant cells were treated with AZD9291 for 72 h, and then cell viabilities were examined by CCK8 assays. **e** and **g** Measurement of antiproliferation effects of AZD9291 by EdU incorporation assay. **f** and **h** Quantitative results of EdU incorporation assay. The numbers of proliferative cells were normalized to that of the control group. All the data were presented as means ± SEM from three independent experiments (*P < 0.05)

The cell counting kit-8 (CCK8) assay to evaluate the effects of the third-generation EGFR inhibitor, AZD9291, on the growth of the six GBM cell lines. Our results showed that AZD9291 significantly inhibited the growth of the six GBM cell lines and demonstrated a dose-dependent inhibitory effect, with IC_50_ values ranging from 1.25 to 3.0 μΜ (Fig. 1a). Consistent with previous reports, our study showed that the IC_50_ of each of the first-generation EGFR inhibitors was above 10 μΜ, and the inhibitory activity of AZD9291 on GBM cell growth was 10-fold higher than that of either of the first-generation EGFR inhibitors (Fig. 1b and c). These results suggested that AZD9291 may be a potential EGFR-targeted drug for GBM treatment and may achieve better therapeutic efficacy than the first-generation EGFR inhibitors can.

To further investigate whether AZD9291 inhibits GBM cells growth due to off-target effect, we constructed two U87 cell lines stably expressing wild-type or Cys797 mutant EGFR, respectively. The Cys797 residue in the catalytic domain of EGFR is essential for the inhibitory effect of AZD9291 [26]. Our data showed that AZD9291 treatment significantly inhibited the growth of the cells expressing wild-type EGFR. However, the inhibitory effects of AZD9291 on the growth of glioma cells were nearly abolished in the cells expressing Cys797 mutant EGFR (Fig. 1d).

To verify the inhibitory effect of AZD9291 on the proliferation of GBM cells, the U87 and U251 GBM cell lines were treated with AZD9291. This was followed by measuring cell proliferation by an EdU assay. As shown in Fig. 1e-h, the AZD9291 treatment groups had significantly lower EdU-positive cell counts than did the control groups of the U87 and U251 GBM cell lines. The percentages of EdU-positive cells in the U87 and U251 GBM cell lines after 2 μΜ AZD9291 treatment were reduced to 25.59 and 37.37%, respectively. These results suggested that AZD9291 significantly inhibited GBM cell proliferation in a dose-dependent manner.

### AZD9291 inhibits colony formation and arrests the GBM cell cycle

**Fig. 2:**
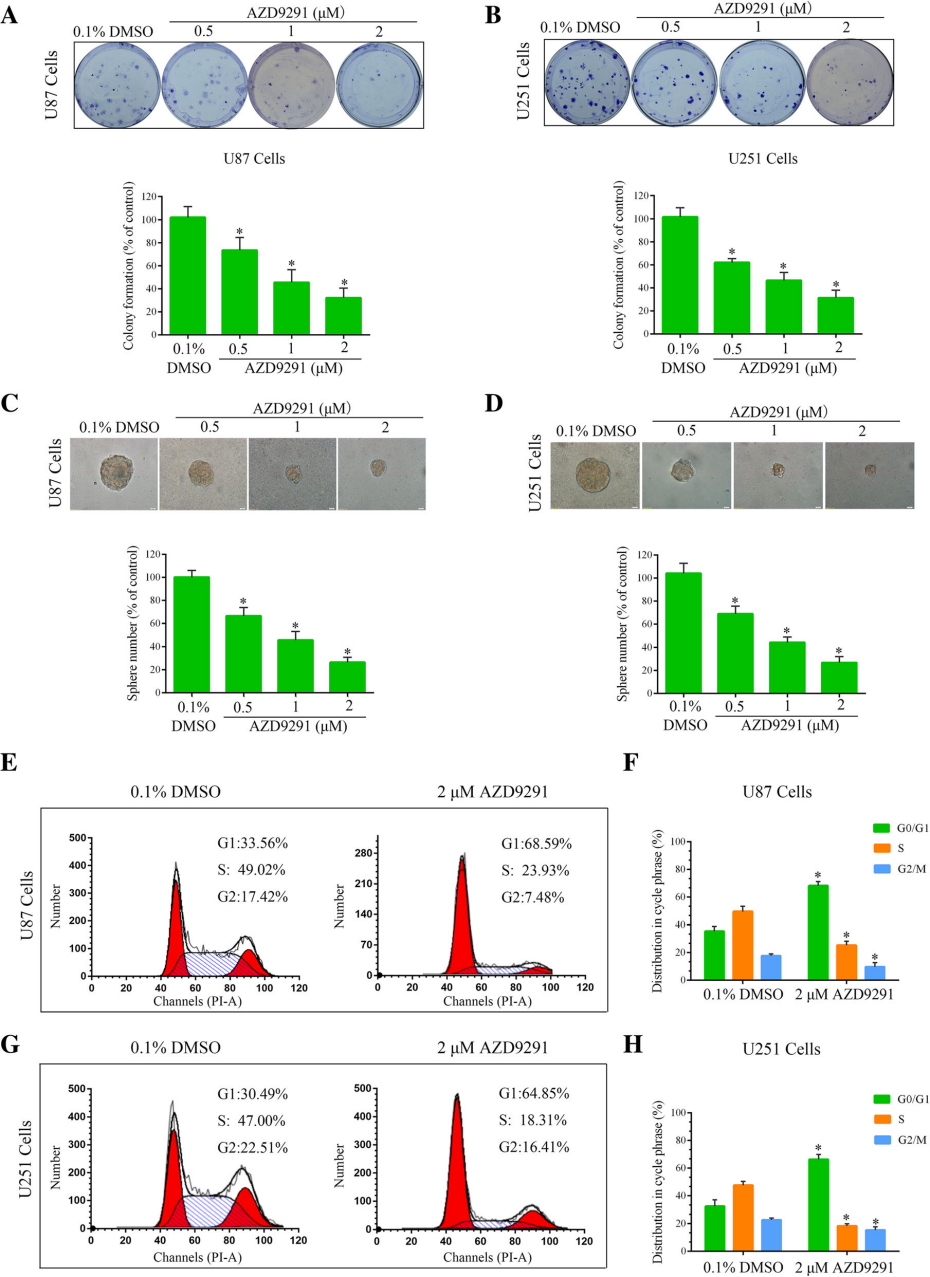
AZD9291 treatment inhibits colony formation and induces cell cycle arrest**. a** and **b** U87 and U251 cells were treated with different concentrations of AZD9291 for 24 h, and then changed with drug-free medium for another 12 day. The numbers of colony formation were counted. The numbers of colony formation were normalized to the control group. **c** and **d** Colony formation abilities were assessed by methylcellulose clonal assays. The number and size of clones were statistically analyzed between the control group and AZD9291 treatment group. **e** and **g** Representative data of the cell cycle analysis of AZD9291-treated cells. U87 and U251 cells were treated with AZD9291 at the indicated concentrations for 24 h. Cell cycle profile was evaluated using flow cytometry. **f** and **h** Quantitative analysis of cell cycle phase distribution in the control group and the AZD9291-treated group. The data from three independent experiments were expressed as the means ± SEM (*P < 0.05)

To observe the long-term inhibitory effect of AZD9291 on the GBM cell cycle, we used a colony formation assay to evaluate the effect of AZD9291 on the abilities of the U87 and U251 GBM cell lines to form colonies. As shown in Fig. 2a and b, AZD9291 significantly inhibited colony formation in these GBM cell lines. Compared with the control group, the number of colonies formed in the U87 cells after 2 μΜ AZD9291 treatment was significantly reduced, by 67.82%. A similar result was also observed in the U251 cells. Subsequently, the methylcellulose colony assay was used to determine the ability of GBM cell lines to create colonies with or without AZD9291 pretreatment. We found that AZD9291 treatment significantly reduced the colony numbers and sizes in U87 and U251 cells (Fig. 2c and d). Taken together, these data indicated that AZD9291 significantly inhibited the ability of GBM cells to form colonies.

To investigate the mechanism of AZD9291 inhibition of GBM cell proliferation, this study used flow cytometry to analyze the cell cycle distribution. As shown in Fig. 2e-h, cell cycle progression was arrested in the G1 phase in both U87 and U251 GBM cell lines after AZD9291 treatment. Compared with untreated cells, the number of cells in the G1 phase after the AZD9291 treatment was significantly increased, and the numbers of cells in the S phase and G2 phase were reduced accordingly. These results indicated that AZD9291-induced reduction of GBM cell proliferation was due to cell cycle arrest in the G1 phase.

### AZD9291 inhibits the migration and invasion of GBM cells

**Fig. 3:**
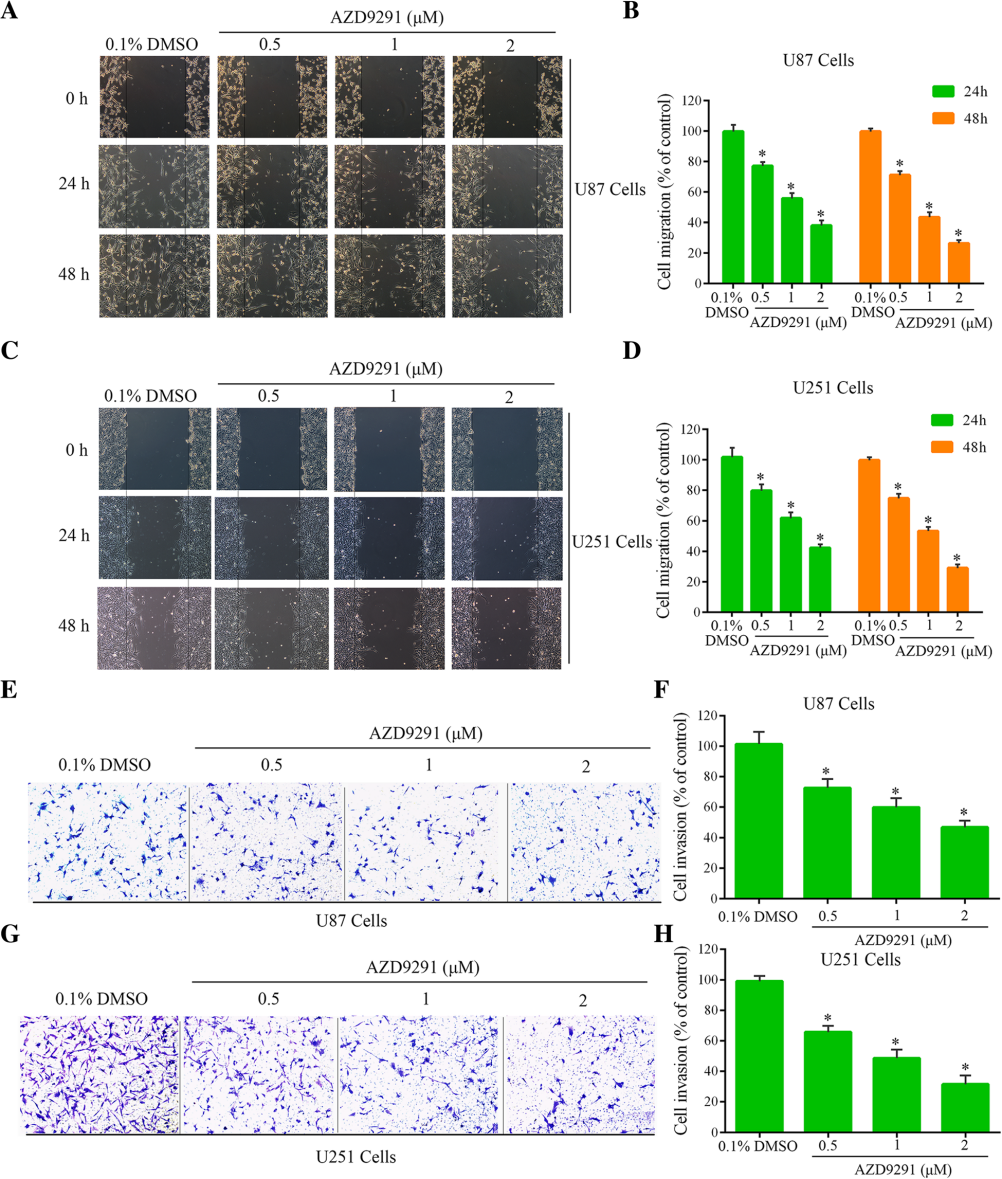
AZD9291 suppressed the migration and invasion of GBM cell. **a** and **c** Cell migratory ability of the control group and the AZD9291-treated groups were detected by wound healing assay in U87 and U251 cells. **b** and **d** Quantitative analysis of migratory cell numbers. **e** and **g** Effects of AZD9291 on invasion ability of U87 and U251 cells as examined by transwell assay. **f** and **h** Quantitative analysis of invading cell numbers. The numbers of migratory and invading cells were normalized to that of the control group. The values were presented as the means ± SEM, *P < 0.05, versus the control

To clarify the effects of AZD9291 on the migration and invasion of GBM cells, we performed wound healing assays and Transwell invasion assays. Compared with the control group, the number of U87 cells that migrated into the scratched region after 24 h and 48 h of AZD9291 (2 μΜ) treatment were significantly reduced to 38 and 26.33%, respectively. U251 cell migration numbers at 24 and 48 h of AZD9291 (2 μΜ) treatment were significantly reduced to 42.33 and 29%, respectively (Fig. 3a-d). In addition, the Transwell assay showed similar results. Compared with the control group, the invasion rates of the U87 and U251 GBM cells after the 2 μΜ AZD9291 treatment were significantly reduced by 53.12 and 68.56%, respectively (Fig. 3e-h). To exclude the possibility that the reduced cell migration number and the reduced cell invasion number are not caused by the inhibitory effect of AZD9291 on cell viability, we pretreated cells with AZD9291, and then we used the treated cells for transwell migration assay and invasion assay. These results further demonstrated that AZD9291 significantly inhibited the migration and invasion of GBM cells in a dose-dependent manner (Additional file 1: Figure S1 and S2).

### AZD9291 inhibits the growth of GBM in an intracranial xenografts mouse model

**Fig. 4:**
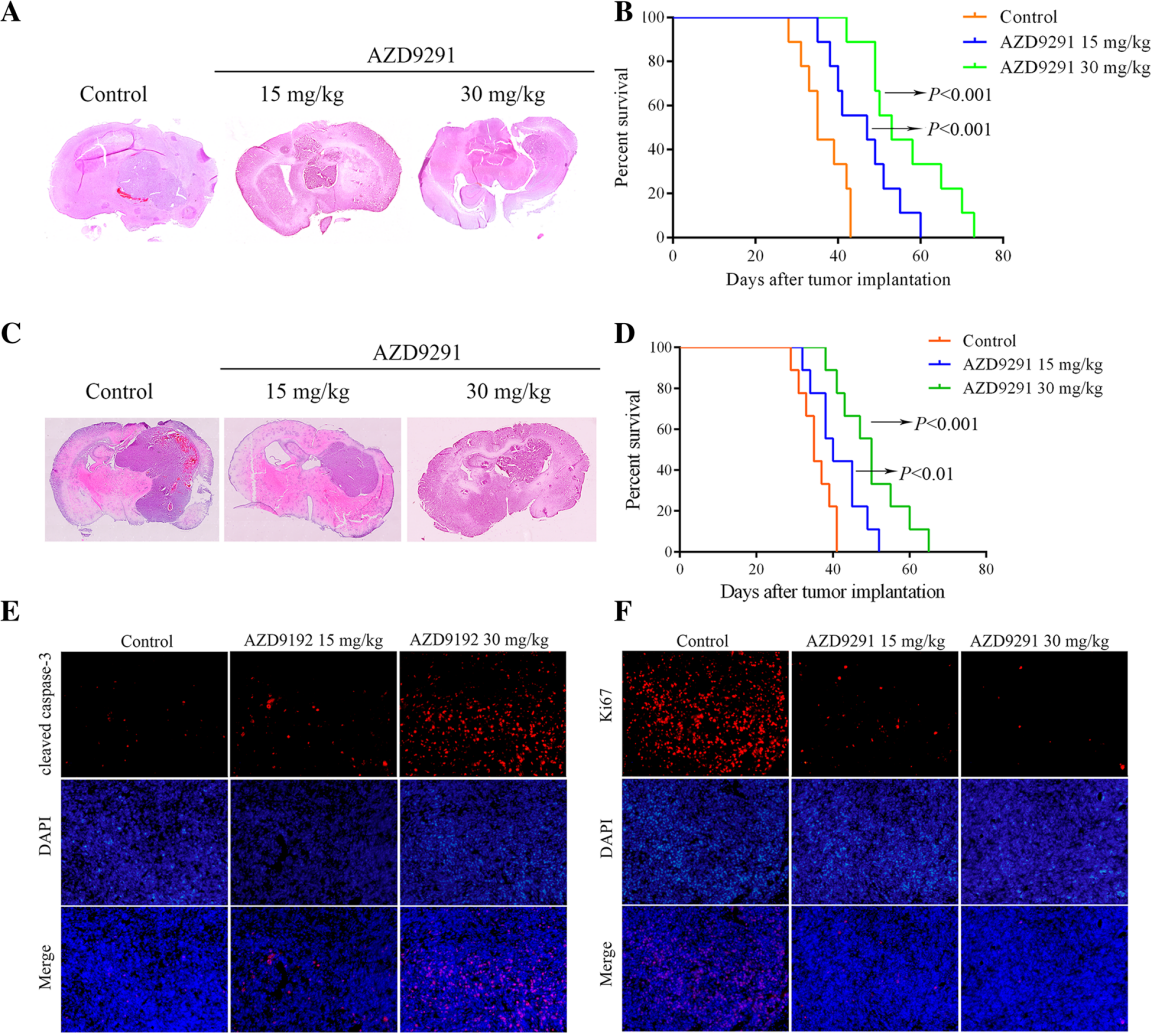
AZD9291 inhibits GBM tumorigenesis in vivo. **a** Mice bearing U87 xenograft tumor were treated with AZD9291 by oral administration. Representative images of H&E staining of whole brain sections from control group and AZD9291 treatment group. **b** The survival analysis of mice with AZD9291 treatment by oral administration. **c** U87 cells were transplanted into the right striatum of nude mice to establish an intracranial GBM model. Mice were sacrificed at 4 weeks after treatment via intraperitoneal injections with vehicle, AZD9291 15 mg/kg and AZD9291 30 mg/kg, respectively. Representative images of H&E staining of coronal sections from mouse brains with orthotopic tumors. **d** The survival of mice with tumors derived from the vehicle or AZD9291-treated groups were measured by Kaplan-Meier survival curves. **e** and **f** The effects of AZD9291 on cell proliferation and apoptosis in vivo. Cell proliferation and apoptosis of the orthotopic tumor were assessed with anti-Ki67 and anti-cleaved caspase-3 immunostaining. Scale bar: 50 μm, **P < 0.01, ***P < 0.001

To determine if AZD9291 also inhibits GBM proliferation in vivo, we established an in situ GBM model in nude mice and analyze the size of the GBM xenografts and the survival of the GBM-bearing mice. The mice were treated with intraperitoneal injection and oral administration of AZD9291, respectively. In oral administration group, the GBM xenografts in AZD9291-treated mice were visibly smaller than those in the vehicle-treated mice (Fig. 4a). Consequently, the AZD9291-treated mice exhibited significantly increased survival (Fig. 4b). In intraperitoneal injection group, AZD9291 treatment significantly inhibited the growth of U87 GBM cells in vivo after 25 d of treatment (Fig. 4c). Survival of the GBM-bearing mice was significantly prolonged after the AZD9291 treatment (Fig. 4d). Further analysis of the effect of AZD9291 on the proliferation and apoptosis of GBM cells in vivo by immunofluorescence staining of the GBM sections with antibodies against Ki67 and cleaved caspase-3 (an apoptotic marker) showed that the numbers of the Ki67-positive cells and the cleaved caspase-3-positive cells in the GBM sections in the AZD9291 treatment group were significantly higher than those in the control group (Fig. 4e and f), suggesting that AZD9291 inhibited the proliferation and promoted apoptosis of the GBM cells in vivo*.*

### AZD9291 continuously inhibits activation of the EGFR/ERK pathway in GBM cells

**Fig. 5:**
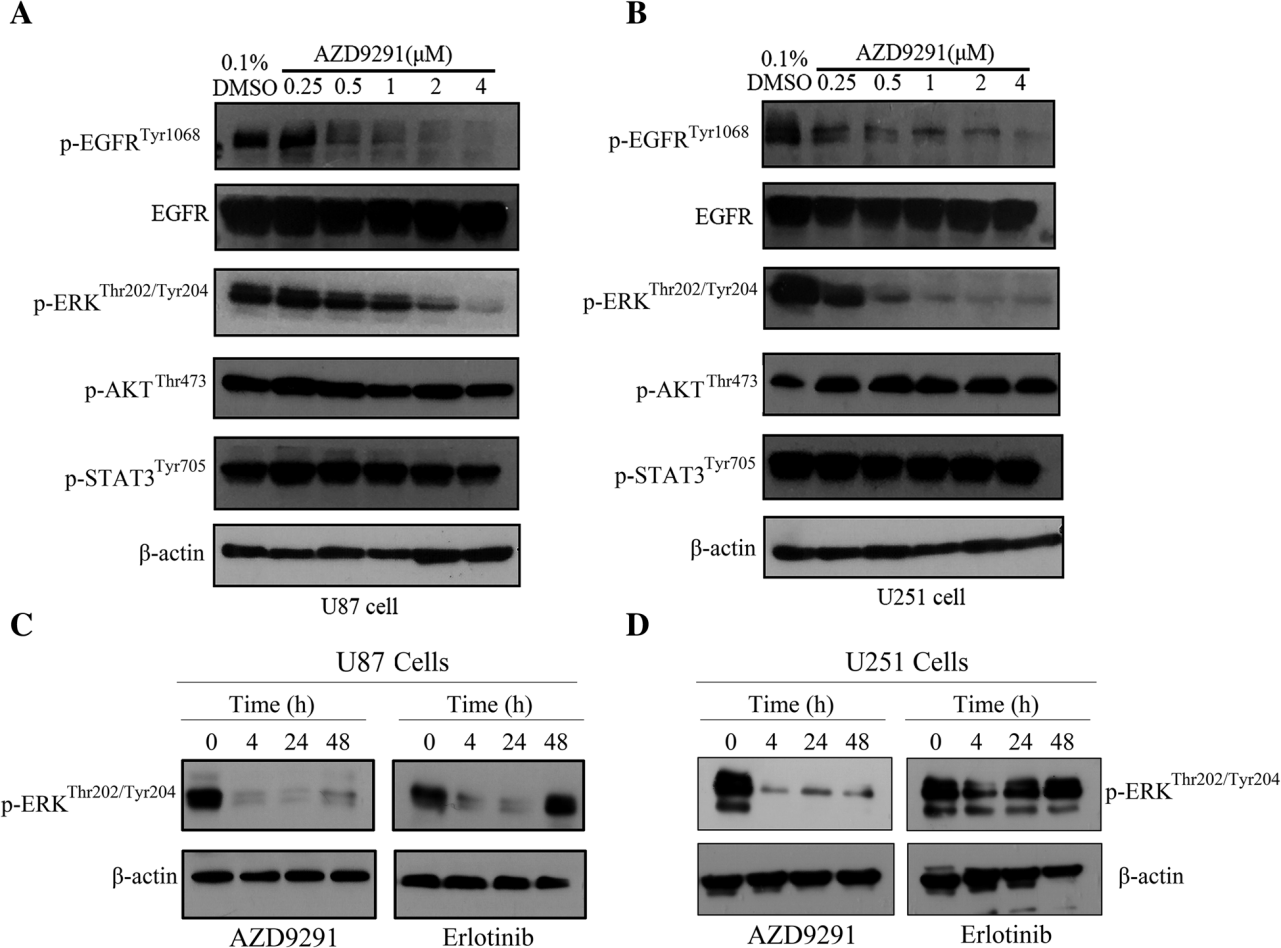
AZD9291 continuously blocks the activation of EGFR/ERK pathway in GBM cells**. a** and **b** U87 and U251 cells were exposed to indicated concentrations of AZD9291 for 24 h and then stimulated with EGF (100 ng/mL) for 30 min. Cell lysates were prepared and examined using Western blot analysis with indicated antibodies. **c** and **d** U87 and U251 cells were treated with AZD9291 or Erlotinib for 4, 24 and 48 h. The expression level of p-ERK were assessed using Western blot analysis

To elucidate the mechanism of AZD9291 inhibition of GBM cell proliferation, western blot analysis was used to evaluate the effect of AZD9291 on EGFR, AKT, STAT3, and ERK phosphorylation in GBM cells. In Fig. 5a and b, different concentrations of AZD9291 treatment in U87 and U251 GBM cells showed had no significant changes in total EGFR expression. However, the expression of phosphorylated EGFR was gradually reduced with increasing AZD9291 concentrations. AZD9291 significantly lowered the phosphorylation level of ERK but had no effect on the phosphorylation levels of AKT and STAT3. Recent report has demonstrated that adaptive activation of the ERK pathway in GBM cells mediates the primary resistance to erlotinib. As shown in Fig. 5c and d, we also observed that erlotinib treatment inhibited ERK phosphorylation in GBM cells for a short time, but a reactivation of ERK was seen at 24–48 h in erlotinib-treated cells. Notably, AZD9291 can continuously suppress the phosphorylation of EGFR and ERK. Therefore, AZD9291 may inhibit the growth of GBM cells by continuously blocking the EGFR/ERK pathway.

### Combined with ERK inhibitor PD098059 enhances the anti-proliferation and anti-invasion activities of AZD9291 in GBM cells

**Fig. 6:**
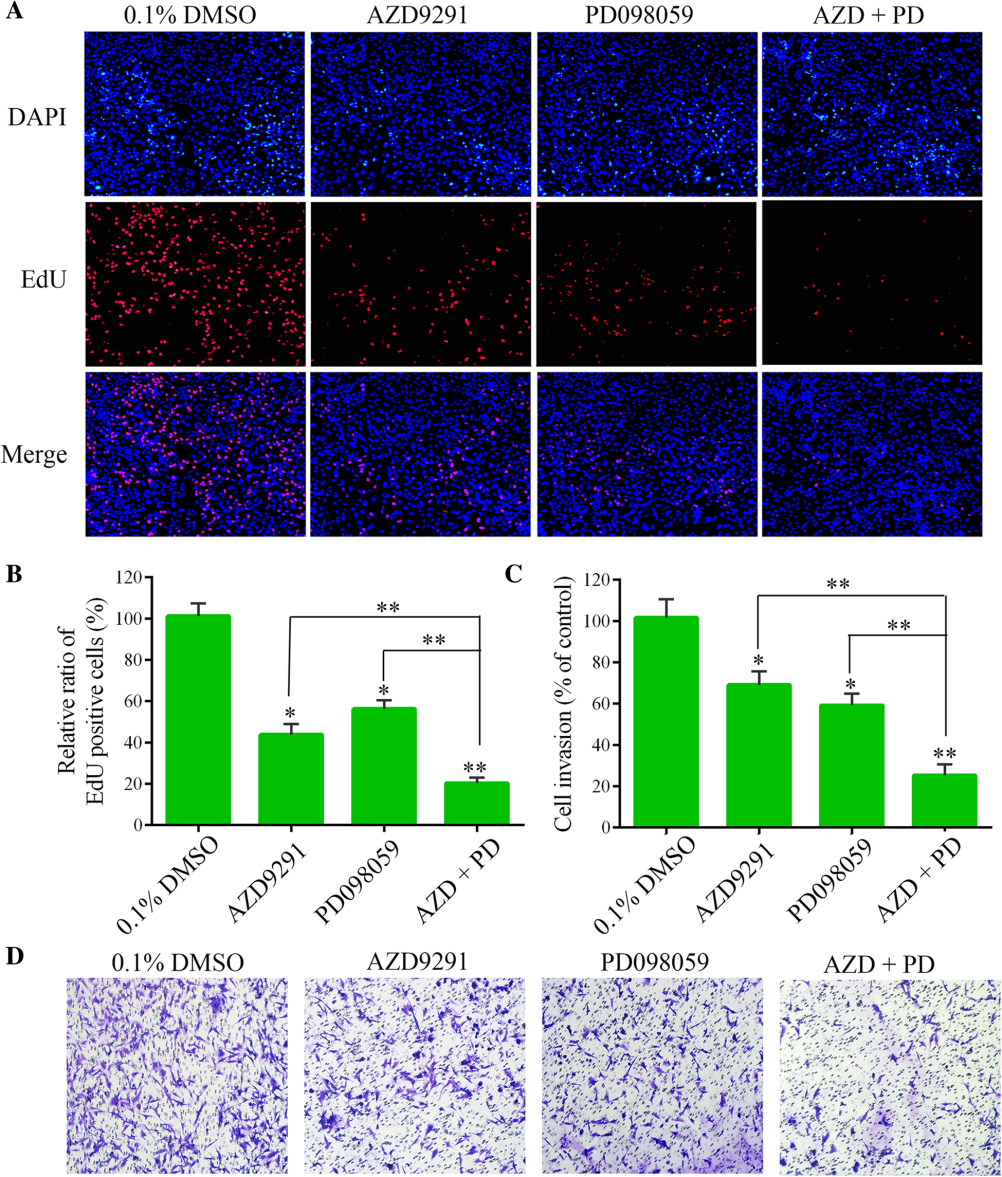
Combined with PD098059, a MEK/ERK inhibitor significantly increases the sensitivity of GBM cells to AZD9291**. a** Measurement of cell proliferation after treating with AZD9291 or PD098059 alone or their combinations by EdU incorporation assay. **b** and **c** Quantitative analysis of proliferative and invading cell numbers. The numbers of proliferative and invading cells were normalized to that of the control group. **d** U251 cells were incubated with AZD9291 or PD098059 alone or their combinations for 30 h. Cell invasive abilities were evaluated by transwell assay. Results were expressed as means ± SEM of three independent experiments. *P < 0.05 and **P < 0.01 compared with control group

We further investigated whether ERK suppression could enhance the inhibitory effect of the AZD9291 on GBM cell function, because AZD9291 inhibit the proliferation of GBM cells by blocking the EGFR/ERK pathway. PD098059 represent specific inhibitor of the ERK cascade [27]. In the EdU assay, we found that either AZD9291 or PD098059 alone inhibited the proliferation of GBM cells. Interestingly, compared with these monotherapies, a combination of AZD9291 and PD098059 significantly further reduced the proportion of EdU-positive cells. A Transwell invasion assay also showed that PD098059 enhanced the inhibitory effect of AZD9291 on GBM cell invasion (Fig. 6). We also combined an ERK inhibitor SCH772984 with AZD9291 to perform EdU proliferation and transwell invasion assays [28]. The combination of AZD9291 with SCH772948 showed synergistic effects on proliferation of GBM cells, but not on cell invasion. (Additional file 1: Figure S3). These data suggested that ERK inhibition could increase the sensitivity of GBM cells to AZD9291.

## Discussion

Although targeted EGFR therapy has achieved significant success in treating epithelial cancers [29], such as lung cancer, its therapeutic effect on GBM has been disappointing. The underlying mechanism of why GBM relies on abnormally activated EGFR pathways but does not respond to EGFR inhibitors has not been fully elucidated. In this study, we showed that the third-generation EGFR inhibitor AZD9291 inhibited the activity of GBM cells > 10-fold more than the first-generation EGFR inhibitors and significantly prolonged survival in a mouse intracranial GBM model.

The first-generation EGFR inhibitors, gefitinib and erlotinib, are competitive reversible inhibitors of ATP binding, as they are ATP analogs [30]. The third-generation EGFR inhibitor, AZD9291, irreversibly inhibits EGFR activity by covalent binding [26, 31]. Thus, this drug has higher activity and selectivity than the first-generation EGFR inhibitors do. Previous reports and our results indicated that the IC_50_ of gefitinib and erlotinib was over 20 μM in GBM cells [32, 33]. Given the challenge of achieving the needed plasma drug concentration in safe doses, it is unlikely that this concentration of gefitinib and erlotinib can be safely applied in the brain. This may be one of the reasons for the failure of the first-generation EGFR inhibitors for the treatment of GBM in clinical trials. However, in this study, the IC_50_ of AZD9291 needed to inhibit U87 and U251 GBM cells was less than 2 μM. Importantly, AZD9291 has shown a good ability to penetrate through the blood-brain barrier. Administration of 25 mg/kg of AZD9291 in mice resulted in 2.98 μM and 7.13 μM concentrations of AZD9291 in the plasma and the brain tissue, respectively [20]. More importantly, a previous clinical trial of AZD9291 treatment in lung cancer patients with brain metastases showed that AZD9291 effectively eliminated the metastatic tumor cells in the brain [21]. In our study, AZD9291 significantly prolonged the survival of mice modeling intracranial GBM. In summary, AZD9291 may be a suitable potential targeted drug for the treatment of GBM because of its properties which are not available in the first- and second-generation EGFR inhibitors, including good activity and ability to penetrate across the blood-brain barrier.

Numerous studies have confirmed that abnormal activation of EGFR is one of the key factors in GBM pathogenesis [34, 35]. In GBM, EGFR primarily regulates the AKT, RAF/ERK, and STAT3 pathways [16, 36, 37]. Extracellular EGFR mutations continuously activate ERK/matrix metallopeptidase 1 (MMP1) signaling and enhance the invasion and proliferation of GBM cells [38]. We found that AZD9291 significantly inhibited the phosphorylation of ERK but had no impact on the phosphorylation levels of AKT and STAT3. These results may be due to other key molecules which regulate AKT and STAT3 activities in GBM. Inhibition of EGFR alone did not completely block AKT and STAT3 activity. The first- and second-generation EGFR inhibitors also mainly inhibited the ERK pathway. Recent report has shown that adaptive activation of the ERK pathway in GBM cells mediates the primary resistance to erlotinib [39]. Erlotinib treatment inhibits ERK phosphorylation in GBM cells for a short time, but ERK phosphorylation was restored 24 h later, possibly reactivated by feedback pathway. Inhibition of the JNK/ERK pathway enhances the sensitivity of GBM cells to erlotinib-mediated inhibition of proliferation. Unlike erlotinib, AZD9291 in this study continuously suppressed the phosphorylation of EGFR and ERK. This may be one of the reasons why AZD9291 inhibited GBM cell proliferation by more than 10-fold more than the first-generation EGFR inhibitors. Our data suggested that AZD9291 might overcome the primary resistance to erlotinib in GBM cells. These results also suggested that the abnormal signaling pathways in GBM cells are very complex, and inhibition of a single pathway may possibly be ineffective. Combination therapy using the third-generation EGFR inhibitor and inhibitor(s) of the AKT and/or STAT3 signaling pathways may be a more effective approach for the treatment of GBM than monotherapy.

## Conclusions

In summary, our study showed that AZD9291 has better ability to inhibit the proliferation of GBM cells than other EGFR-TKIs. AZD9291 significantly prolonged the survival of our preclinical GBM mouse model. The underlying mechanism for this may be that AZD9291 inhibited GBM cell proliferation by sustained and efficient blocking of the EGFR/ERK signaling pathway. Given its very good permeability of the blood-brain barrier, the approval of clinical applications for the treatment of lung cancer, and its good safety and tolerance, AZD9291 is supported by our findings as a good choice to be applied in future clinical trials for the treatment of GBM, either as monotherapy or combined with other drugs.

## Additional file

Additional file 1: **Figure S1.** AZD9291 pretreatment inhibited GBM cell migration. The cells were pretreated with AZD9291 for 24 h, and then the treated cells were used to conduct transwell migration assay (no Matrigel). Representative images of migratory abilities of AZD929-treated U251cells (A) and U87 cells (B). **Figure S2.** AZD9291 pretreatment inhibited GBM cell invasion. The cells were pretreated with AZD9291 for 24 h, and then the treated cells were used to conduct transwell invasion assays (Matrigel was added). Representative images of invasive abilities of AZD929-treated U251cells (A) and U87 cells (B). **Figure S3.** Combined with SCH772984, another ERK inhibitor significantly increases the sensitivity of GBM cells to AZD9291. (A and B) Measurement of cell proliferation after treating with AZD9291 or SCH772984 alone or their combinations by EdU incorporation assay. (C-F) U87 and U251 cells were incubated with AZD9291 or SCH772984 alone or their combinations for 30 h. Cell invasive abilities were evaluated by transwell invasion assay. (DOCX 3630 kb)

## Abbreviations

CCK-8: Cell counting kit-8
DAPI: 4′,6-diamidino-2-phenylindole
EdU: 5-ethynyl-2′-deoxyuridine
EGF: Epidermal growth factor
EGFR: Epidermal growth factor receptor
GBM: Glioblastoma multiforme

## References

[1] Nduom EK, Wei J, Yaghi NK, Huang N, Kong LY, Gabrusiewicz K (2016). PD-L1 expression and prognostic impact in glioblastoma. Neuro-Oncology.

[2] Liu X, Chong Y, Tu Y, Liu N, Yue C, Qi Z (2016). CRM1/XPO1 is associated with clinical outcome in glioma and represents a therapeutic target by perturbing multiple core pathways. J Hematol Oncol.

[3] Prados MD, Byron SA, Tran NL, Phillips JJ, Molinaro AM, Ligon KL (2015). Toward precision medicine in glioblastoma: the promise and the challenges. Neuro-Oncology.

[4] Westover D, Zugazagoitia J, Cho BC, Lovly CM, Paz-Ares L (2018). Mechanisms of acquired resistance to first- and second-generation EGFR tyrosine kinase inhibitors. Ann Oncol.

[5] Brennan CW, Verhaak RG, McKenna A, Campos B, Noushmehr H, Salama SR (2013). The somatic genomic landscape of glioblastoma. Cell..

[6] Eskilsson E, Rosland GV, Solecki G, Wang Q, Harter PN, Graziani G (2018). EGFR heterogeneity and implications for therapeutic intervention in glioblastoma. Neuro-Oncology.

[7] Vivanco I, Robins HI, Rohle D, Campos C, Grommes C, Nghiemphu PL (2012). Differential sensitivity of glioma- versus lung cancer-specific EGFR mutations to EGFR kinase inhibitors. Cancer Discov..

[8] Padfield E, Ellis HP, Kurian KM (2015). Current therapeutic advances targeting EGFR and EGFRvIII in glioblastoma. Front Oncol.

[9] Li L, Puliyappadamba VT, Chakraborty S, Rehman A, Vemireddy V, Saha D (2015). EGFR wild type antagonizes EGFRvIII-mediated activation of met in glioblastoma. Oncogene..

[10] Brandes AA, Franceschi E, Tosoni A, Hegi ME, Stupp R (2008). Epidermal growth factor receptor inhibitors in neuro-oncology: hopes and disappointments. Clin Cancer Res.

[11] Thorne AH, Zanca C, Furnari F (2016). Epidermal growth factor receptor targeting and challenges in glioblastoma. Neuro-Oncology.

[12] Chakravarti A, Wang M, Robins HI, Lautenschlaeger T, Curran WJ, Brachman DG (2013). RTOG 0211: a phase 1/2 study of radiation therapy with concurrent gefitinib for newly diagnosed glioblastoma patients. Int J Radiat Oncol Biol Phys.

[13] Prados MD, Chang SM, Butowski N, DeBoer R, Parvataneni R, Carliner H (2009). Phase II study of erlotinib plus temozolomide during and after radiation therapy in patients with newly diagnosed glioblastoma multiforme or gliosarcoma. J Clin Oncol.

[14] Sathornsumetee S, Desjardins A, Vredenburgh JJ, McLendon RE, Marcello J, Herndon JE (2010). Phase II trial of bevacizumab and erlotinib in patients with recurrent malignant glioma. Neuro-Oncology.

[15] Reardon DA, Nabors LB, Mason WP, Perry JR, Shapiro W, Kavan P (2015). Phase I/randomized phase II study of afatinib, an irreversible ErbB family blocker, with or without protracted temozolomide in adults with recurrent glioblastoma. Neuro-Oncology.

[16] Kwatra MM (2017). A rational approach to target the epidermal growth factor receptor in glioblastoma. Curr Cancer Drug Targets.

[17] Janne PA, Yang JC, Kim DW, Planchard D, Ohe Y, Ramalingam SS (2015). AZD9291 in EGFR inhibitor-resistant non-small-cell lung cancer. N Engl J Med.

[18] Butterworth S, Cross DAE, Finlay MRV, Ward RA, Waring MJ (2017). The structure-guided discovery of osimertinib: the first U.S. FDA approved mutant selective inhibitor of EGFR T790M. Medchemcomm..

[19] Wang J, Gan C, Sparidans RW, Wagenaar E, van Hoppe S, Beijnen JH (2018). P-glycoprotein (MDR1/ABCB1) and breast Cancer resistance protein (BCRP/ABCG2) affect brain accumulation and intestinal disposition of encorafenib in mice. Pharmacol Res.

[20] Ballard P, Yates JW, Yang Z, Kim DW, Yang JC, Cantarini M (2016). Preclinical comparison of Osimertinib with other EGFR-TKIs in EGFR-mutant NSCLC brain metastases models, and early evidence of clinical brain metastases activity. Clin Cancer Res.

[21] Cross DA, Ashton SE, Ghiorghiu S, Eberlein C, Nebhan CA, Spitzler PJ (2014). AZD9291, an irreversible EGFR TKI, overcomes T790M-mediated resistance to EGFR inhibitors in lung cancer. Cancer Discov..

[22] Finlay MR, Anderton M, Ashton S, Ballard P, Bethel PA, Box MR (2014). Discovery of a potent and selective EGFR inhibitor (AZD9291) of both sensitizing and T790M resistance mutations that spares the wild type form of the receptor. J Med Chem.

[23] Niu M, Xu X, Shen Y, Yao Y, Qiao J, Zhu F (2015). Piperlongumine is a novel nuclear export inhibitor with potent anticancer activity. Chem Biol Interact.

[24] Yue C, Niu M, Shan QQ, Zhou T, Tu Y, Xie P (2017). High expression of Bruton's tyrosine kinase (BTK) is required for EGFR-induced NF-kappaB activation and predicts poor prognosis in human glioma. J Exp Clin Cancer Res.

[25] Niu M, Cai W, Liu H, Chong Y, Hu W, Gao S (2015). Plumbagin inhibits growth of gliomas in vivo via suppression of FOXM1 expression. J Pharmacol Sci.

[26] Thress KS, Paweletz CP, Felip E, Cho BC, Stetson D, Dougherty B (2015). Acquired EGFR C797S mutation mediates resistance to AZD9291 in non-small cell lung cancer harboring EGFR T790M. Nat Med.

[27] Tang H, Zhao J, Zhang L, Zhao J, Zhuang Y, Liang P (2016). SRPX2 enhances the epithelial-mesenchymal transition and Temozolomide resistance in glioblastoma cells. Cell Mol Neurobiol.

[28] Hayes TK, Neel NF, Hu C, Gautam P, Chenard M, Long B (2016). Long-term ERK inhibition in KRAS-mutant pancreatic Cancer is associated with MYC degradation and senescence-like growth suppression. Cancer Cell.

[29] Soria JC, Ohe Y, Vansteenkiste J, Reungwetwattana T, Chewaskulyong B, Lee KH (2018). Osimertinib in untreated EGFR-mutated advanced non-small-cell lung Cancer. N Engl J Med.

[30] Pao W, Chmielecki J (2010). Rational, biologically based treatment of EGFR-mutant non-small-cell lung cancer. Nat Rev Cancer.

[31] Yosaatmadja Y, Silva S, Dickson JM, Patterson AV, Smaill JB, Flanagan JU (2015). Binding mode of the breakthrough inhibitor AZD9291 to epidermal growth factor receptor revealed. J Struct Biol.

[32] Chang CY, Kuan YH, Ou YC, Li JR, Wu CC, Pan PH (2014). Autophagy contributes to gefitinib-induced glioma cell growth inhibition. Exp Cell Res.

[33] Pedeboscq S, L'Azou B, Passagne I, De Giorgi F, Ichas F, Pometan JP (2008). Cytotoxic and apoptotic effects of bortezomib and gefitinib compared to alkylating agents on human glioblastoma cells. J Exp Ther Oncol.

[34] Ma Y, Tang N, Thompson RC, Mobley BC, Clark SW, Sarkaria JN (2016). InsR/IGF1R pathway mediates resistance to EGFR inhibitors in glioblastoma. Clin Cancer Res.

[35] Furnari FB, Cloughesy TF, Cavenee WK, Mischel PS (2015). Heterogeneity of epidermal growth factor receptor signalling networks in glioblastoma. Nat Rev Cancer.

[36] Tanaka K, Babic I, Nathanson D, Akhavan D, Guo D, Gini B (2011). Oncogenic EGFR signaling activates an mTORC2-NF-kappaB pathway that promotes chemotherapy resistance. Cancer Discov.

[37] Liu F, Hon GC, Villa GR, Turner KM, Ikegami S, Yang H (2015). EGFR mutation promotes glioblastoma through epigenome and transcription factor network remodeling. Mol Cell.

[38] Binder ZA, Thorne AH, Bakas S, Wileyto EP, Bilello M, Akbari H (2018). Epidermal growth factor receptor extracellular domain mutations in glioblastoma present opportunities for clinical imaging and therapeutic development. Cancer Cell.

[39] Guo G, Gong K, Ali S, Ali N, Shallwani S, Hatanpaa KJ (2017). A TNF-JNK-Axl-ERK signaling axis mediates primary resistance to EGFR inhibition in glioblastoma. Nat Neurosci.

