# Supplementary material for: The third-generation EGFR inhibitor AZD9291 overcomes primary resistance by continuously blocking ERK signaling in glioblastoma
Source: J Exp Clin Cancer Res. 2019 May 23;38:219. doi: 10.1186/s13046-019-1235-7 (PMC6533774; doi:10.1186/s13046-019-1235-7)
Supplement: Supplementary file 1 — Figure S1. AZD9291 pretreatment inhibited GBM cell migration. The cells were pretreated with AZD9291 for 24 h, and then the treated cells were used to conduct transwell migration assay (no Matrigel). Representative images of migratory abilities of AZD929-treated U251cells (A) and U87 cells (B). Figure S2. AZD9291 pretreatment inhibited GBM cell invasion. The cells were pretreated with AZD9291 for 24 h, and then the treated cells were used to conduct transwell invasion assays (Matrigel was added). Representative images of invasive abilities of AZD929-treated U251cells (A) and U87 cells (B). Figure S3. Combined with SCH772984, another ERK inhibitor significantly increases the sensitivity of GBM cells to AZD9291. (A and B) Measurement of cell proliferation after treating with AZD9291 or SCH772984 alone or their combinations by EdU incorporation assay. (C-F) U87 and U251 cells were incubated with AZD9291 or SCH772984 alone or their combinations for 30 h. Cell invasive abilities were evaluated by transwell invasion assay. (DOCX 3630 kb) [file 13046_2019_1235_MOESM1_ESM.docx]

**Additional file 1**


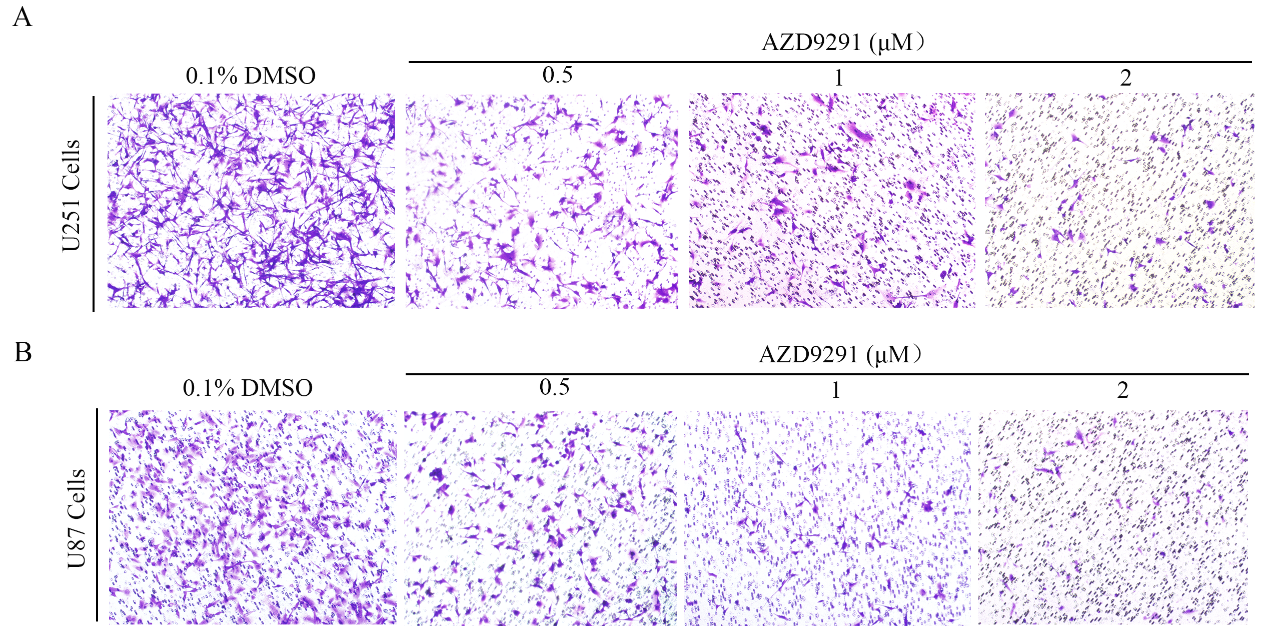


**Figure S1. AZD9291 pretreatment inhibited GBM cell migration**. The cells were pretreated with AZD9291 for 24 h, and then the treated cells were used to conduct transwell migration assay (no Matrigel). Representative images of migratory abilities of AZD929-treated U251cells (A) and U87 cells (B).


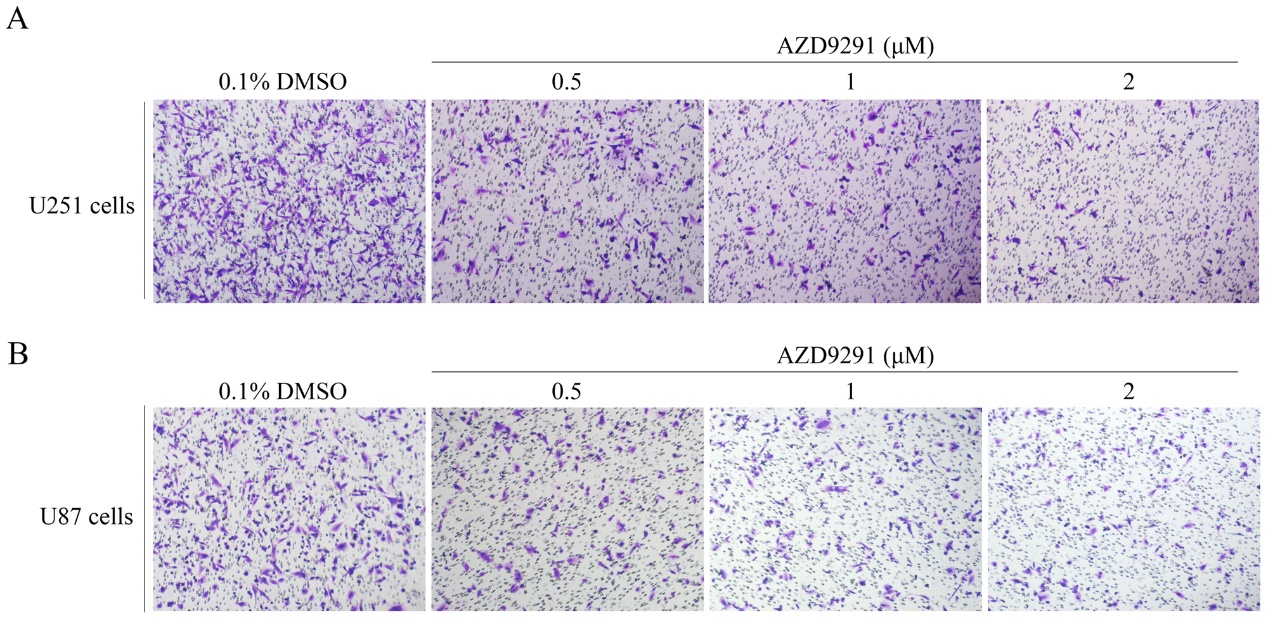


**Figure S2. AZD9291 pretreatment inhibited GBM cell invasion**. The cells were pretreated with AZD9291 for 24 h, and then the treated cells were used to conduct transwell invasion assays (Matrigel was added). Representative images of invasive abilities of AZD929-treated U251cells (A) and U87 cells (B).


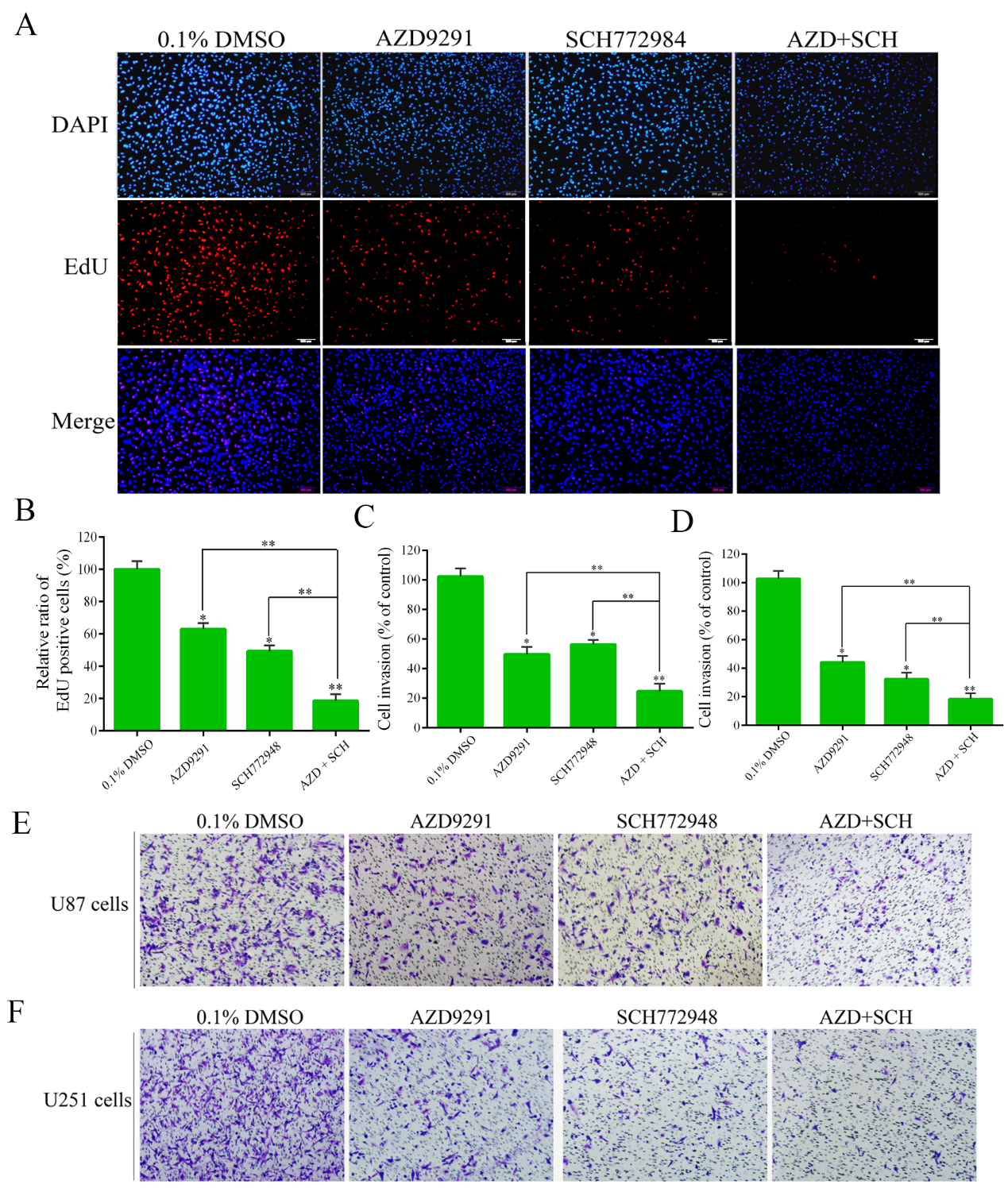


**Figure S3.** **Combined with SCH772984, another ERK inhibitor significantly increases the sensitivity of GBM cells to AZD9291.** (A and B) Measurement of cell proliferation after treating with AZD9291 or SCH772984 alone or their combinations by EdU incorporation assay. (C-F) U87 and U251 cells were incubated with AZD9291 or SCH772984 alone or their combinations for 30 h. Cell invasive abilities were evaluated by transwell invasion assay.
